# Sequential Analysis of Binding and Neutralizing Antibody in COVID-19 Convalescent Patients at 14 Months After SARS-CoV-2 Infection

**DOI:** 10.3389/fimmu.2021.793953

**Published:** 2021-11-26

**Authors:** Margherita Rosati, Evangelos Terpos, Ioannis Ntanasis-Stathopoulos, Mahesh Agarwal, Jenifer Bear, Robert Burns, Xintao Hu, Eleni Korompoki, Duncan Donohue, David J. Venzon, Meletios-Athanasios Dimopoulos, George N. Pavlakis, Barbara K. Felber

**Affiliations:** ^1^ Human Retrovirus Section, Vaccine Branch, Center for Cancer Research, National Cancer Institute at Frederick, Frederick, MD, United States; ^2^ Department of Clinical Therapeutics, School of Medicine, National and Kapodistrian University of Athens, Athens, Greece; ^3^ Human Retrovirus Pathogenesis Section, Vaccine Branch, Center for Cancer Research, National Cancer Institute at Frederick, Frederick, MD, United States; ^4^ MS Applied Information and Management Sciences, Frederick National Laboratory for Cancer Research, Frederick, MD, United States; ^5^ Biostatistics and Data Management Section, Center for Cancer Research, National Cancer Institute, National Institutes of Health, Bethesda, MD, United States

**Keywords:** longevity, COVID-19, antibody kinetics, SARS-CoV-2, anamnestic response, re-exposure, variants of concern

## Abstract

Durability of SARS-CoV-2 Spike antibody responses after infection provides information relevant to understanding protection against COVID-19 in humans. We report the results of a sequential evaluation of anti-SARS-CoV-2 antibodies in convalescent patients with a median follow-up of 14 months (range 12.4-15.4) post first symptom onset. We report persistence of antibodies for all four specificities tested [Spike, Spike Receptor Binding Domain (Spike-RBD), Nucleocapsid, Nucleocapsid RNA Binding Domain (N-RBD)]. Anti-Spike antibodies persist better than anti-Nucleocapsid antibodies. The durability analysis supports a bi-phasic antibody decay with longer half-lives of antibodies after 6 months and antibody persistence for up to 14 months. Patients infected with the Wuhan (WA1) strain maintained strong cross-reactive recognition of Alpha and Delta Spike-RBD but significantly reduced binding to Beta and Mu Spike-RBD. Sixty percent of convalescent patients with detectable WA1-specific NAb also showed strong neutralization of the Delta variant, the prevalent strain of the present pandemic. These data show that convalescent patients maintain functional antibody responses for more than one year after infection, suggesting a strong long-lasting response after symptomatic disease that may offer a prolonged protection against re-infection. One patient from this cohort showed strong increase of both Spike and Nucleocapsid antibodies at 14 months post-infection indicating SARS-CoV-2 re-exposure. These antibodies showed stronger cross-reactivity to a panel of Spike-RBD including Beta, Delta and Mu and neutralization of a panel of Spike variants including Beta and Gamma. This patient provides an example of strong anti-Spike recall immunity able to control infection at an asymptomatic level. Together, the antibodies from SARS-CoV-2 convalescent patients persist over 14 months and continue to maintain cross-reactivity to the current variants of concern and show strong functional properties.

## Introduction

Understanding the longevity of antibody responses against the Severe Acute Respiratory Syndrome Coronavirus 2 (SARS-CoV-2) is important because it will allow to compare and to contrast infection-induced and vaccination-induced immune responses. We have reported on a sequential analysis of SARS-CoV-2 antibody responses in COVID-19 convalescent patients over a period of 8 months post symptom onset (1, 2). We previously reported a bi-phasic decline with an inflection point at 6 months post symptom onset (1). The shorter half-lives of Spike and Nucleocapsid antibodies during the first 6 months post symptom onset were followed by longer half-lives for both specificities thereafter. In our 8-month follow-up, we also reported that 76% of the patients still had detectable Neutralizing Antibodies (NAb) (1). Many studies reported short-term follow-up observations of less than 6 months (3–15), whereas others reported longitudinal observations, typically spanning less than 12 months post symptom onset (1, 4, 16–27). Some studies reported contraction of anti-COVID humoral responses with a stronger initial decline (1, 4, 23–25, 27).

The Athens cohort of convalescent patients who donated plasma for transfer to acute patients provided an opportunity to perform a sequential analysis up to 14 months post symptom onset using the same methodology of measuring binding and neutralizing antibodies and to characterize their cross-reactivity to a panel of current Spike variants of concern (VOC).

## Materials and Methods

### Study Design

This study included plasma donors who participated in a phase 2 study (NCT04408209 and NCT04743388) for the use of convalescent plasma for the treatment of COVID-19 infection, started in Greece on April 28, 2020. Continued measurements of anti-SARS-CoV-2 binding and neutralizing antibodies (NAb) is a secondary endpoint of the phase 2 study. Donors gave informed consent, as previously described (2). All study procedures were carried out in accordance with the declaration of Helsinki (18^th^ World Medical Association Assembly), its subsequent amendments, the Greek regulations and guidelines, as well as the Good Clinical practice Guidelines (GCP) as defined by the International Conference of Harmonization. The study was approved by the local ethics committees of all participating hospitals.

### Detection of Anti-SARS-CoV2 Antibodies

**Figure 1:**
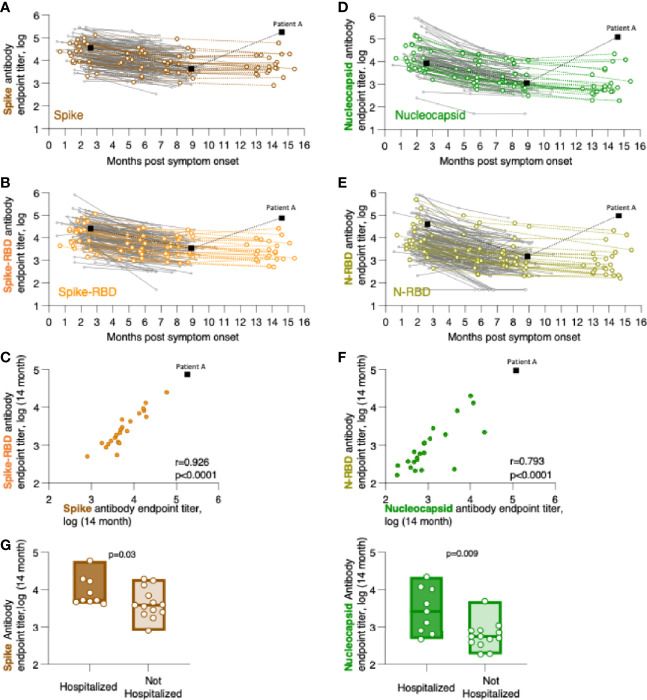
Persistence of SARS-CoV-2 binding antibodies for up to 14 months of follow-up. Binding Ab levels were measured by in-house ELISA using serial dilutions of serum samples and were expressed as endpoint titers (log-transformed). ELISA assays measured antibodies recognizing **(A)** trimeric Spike, **(B)** Spike Receptor Binding Domain (Spike-RBD), **(D)** complete Nucleocapsid, or **(E)** Nucleocapsid RNA Binding Domain (N-RBD). **(C, F)** Relation of CoV-2 antibody levels measured the 14-month time point with correlations of **(C)** Spike and Spike-RBD and **(F)** Nucleocapsid and N-RBD antibodies. Spearman r and p values are given. **(G)** Comparison of association of anti-Spike (left panel) and anti-Nucleocapsid (right panel) antibodies in hospitalized and not hospitalized patients at the 14-month follow-up. The p values (Mann-Whitney) are given.

**Figure 2:**
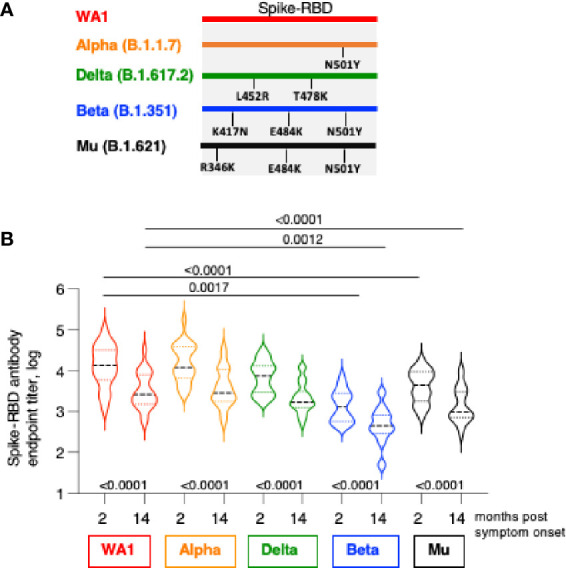
Cross-recognition Spike-RBD variants by SARS CoV-2 infection-induced antibodies. **(A)** The cartoon depicts AA changes in RBD (AA 318-541). Beta and Gamma Spike share the identical RBD but differ in several AA within Spike used in neutralization assay. **(B)** Antibodies were measured by ELISA at 2 and at 14 months post symptom onset using the panel of the indicated Spike-RBD proteins. The p values between were determinated with the Wilcoxon matched-pairs signed rank test; while the comparison between WA1 and variants at month 2 and month 14 were derived using ANOVA Friedman/Dunn’s multiple comparison test. Dotted lines, comparison to WA1 at 2 month; solid lines, comparison to WA1 at 14 month post infection.

For the detection of anti-SARS-CoV-2 antibodies, in-house ELISA assays were used to evaluate 23 sequentially isolated samples at 14 months post symptom onset. Endpoint titer antibodies were determined against trimeric WA1 Spike, Spike Receptor Binding Domain (Spike-RBD) of WA1 (AA 319-529), Alpha (B.1.1.7) (AA 318-529), Beta (B.1.351) (AA 319-541), Delta (B.1.617.2) (AA 318-529) and Mu (AA 318-529); WA1 Nucleocapsid, and WA1 Nucleocapsid RNA Binding Domain (N-RBD) (AA 47-173). Antibody levels were measured using eight 4-fold serial serum dilutions starting at 1:50. Antibody endpoint titers were determined using the cut-off values determined using 17-23 healthy human plasma samples collected between 2015-2018 tested against the different antigens as described (1, 2, 28). If a sample has an endpoint titer of 1:50, it is considered below threshold of the assay and the value is entered as 0.1. A modelfit approach was conducted in R to model the curve to more define endpoint titers (1) shown in **Figure 1**. GraphPad Prism area-under-the-curve was used to determine the endpoint titers above the baseline using lastX feature shown in **Figures 2**, **4**.

### SARS-CoV-2 Pseudotype Neutralization Assay

Neutralization assays were performed using SARS-CoV-2 pseudotyped viruses, as previously described (1, 2, 29). Briefly, serial dilutions (4x fold serial dilutions starting at 1:10) of heat-inactivated sera were incubated with an equal volume of the pseudotyped virions (pHIV_NL_Env-Nanoluc (30, 31) and the virion-Ab mixture was used to transduce HEK293T/ACE2wt cells. The reporter virus was pseudotyped with the Spike spanning 1254 amino acids from WA1, Alpha, Beta and Delta (32). The highest serum concentration analyzed was a 1:40 dilution (a four-fold serial dilution up to 1:655,360). Two days later, the luciferase levels were measured in the cell extracts as ID50 (50% Inhibitory Dose) calculated using GraphPad Prism version 9.2 for MacOS X (GraphPad Software, Inc, La Jolla, CA). The NAb ID50 threshold of quantification in this assay is 0.5 log and the threshold of detection is 0.1 log.

### Statistical Analysis

Slopes calculated between consecutive log(titer) values were tested for the null hypothesis of no change using the one-sample Wilcoxon signed rank test. Half-life estimates were calculated from median slopes. Differences between slopes from consecutive intervals were also tested with the signed rank test. The fit model to deterrmine both the antibody endpoint titers and 50% titers of the neutralization curves were previously described (32) and were determinated using R methods-package (https://www.rdocumentation.org/packages/stats/versions/3.6.2/topics/nls) (1). Analysis was performed by using GraphPad Prism Version 9.2 for MacOS X (GraphPad Software, Inc, La Jolla, CA). Comparisons between the timepoints were made using either ANOVA Friedman/Dunn’s multiple comparison test or Wilcoxon matched-pairs signed rank test.

## Results

### Patient Cohort at 14 Months Past SARS-CoV-2 Infection

We enrolled a cohort of SARS-CoV-2 infected patients who reported symptom onset between March and May 2020 and donated convalescent plasma two months later (1, 2). These patients were followed until July 2021 (**Table 1**) in this study (NCT04408209 and NCT04743388), which started in Greece on April 28, 2020. The study design allowed for the measurements and the sequential evaluation of anti-SARS-CoV-2 antibodies against Spike and Nucleocapsid collected longitudinally over 14 months post symptom onset. We previously reported the results of anti-SARS-CoV-2 antibody levels in 148 convalescent patients covering a median of 2.1 to 8.3 months post infection (1, 2) (**Table 1**). Here, we report an additional follow-up of 23 of these patients remaining un-vaccinated up to a median of 14 months post symptom onset. The other patients were vaccinated before the 14- month time point or were lost to follow-up.

**Table 1:** Characteristics of the study cohort (n=23).

Variables	
Gender	n (%)
*Female*	12 (52%)
*Male*	11 (48%)
Age (years)	n (%): Median [range]
*<50*	14 (61%); 39.5 [27-49]
*≥50*	9 (39%): 57 [50-75]
Hospitalization	n (%)
*no*	13 (57%)
*yes*	10 (43%)
First measurement (screening)	Median [range]
Time since symptom onset (months)	1.9 [0.7-3]
Second measurement (6-month value)	Median [range]
Time since symptom onset (months)	5.7 [2.9-6]
Third measurement (8-month value)	Median [range]
Time since symptom onset (months)	8.1 [7.2-9]
Fourth measurement (14-month value)	Median [range]
Time since symptom onset (months)	13.8 [12.4-15.4]

The 23 patients of the cohort tested were described in **Table 1** regarding age, gender, and COVID-19-related hospitalization. Fourteen patients were 27-49 years old, whereas 9 patients were 50-75 years old. Twelve patients were female and eleven patients were male. Thirteen of the patients did not need hospitalization and 10 had been hospitalized. The composition of this subgroup reflects the characteristics of the originally described 148 patients (1) shown in **Table 2**.

**Table 2:** Comparison of patient cohort from the 8- and 14-month follow-up.

Variables	8-month^1^	14-month^2^
Number of patients	148	23
Gender	n (%)	n (%)
*Female*	71 (48%)	12 (52%)
*Male*	77 (52%)	11 (48%)
Age (years)	n (%):	n (%):
*<50*	72 (49%)	14 (61%)
*≥50*	76 (51%)	9 (39%)
Hospitalization	n (%)	n (%)
*no*	91 (61.5%)	13 (57%)
*yes*	57 (38.5%)	10 (43%)

^1^Patient cohort (n=148) described in Terpos et al. (1).

^2^Patient cohort (n=23) from **Table 1**.

### Durability of Anti-Spike and Anti-Nucleocapsid Antibodies Upon SARS-CoV-2 Infection

We measured the anti-Spike and anti-Nucleocapsid antibody responses to the original Wuhan-HA-1 virus (referred to as WA1) antigens at the 14-month time point (**Figure 1** and **Table 3**). **Figure 1** shows the plots of the previously reported patients (grey lines) up to month 8 post symptom onset overlayed with the measurements (in color) of the 23 patients up to the 14-month time point. We found robust levels of anti-Spike (**Figure 1A**) and anti-Spike-RBD antibodies (**Figure 1B**), with median log endpoint titers of 3.69 [IQR 0.67] and 3.37 [IQR 0.78], respectively (**Table 3**). We also found a strong correlation (Spearman r=0.93, p<0.0001) of anti-Spike and anti-Spike-RBD antibodies (**Figure 1C**), supporting the notion of a continued relation of these specificities, as we reported for the time points of 8 months (1). Of note, one patient (patient A), denoted with black square in **Figure 1**, showed a strong increase in all the antibodies measured as described in detail below (**Figure 4**).

**Table 3:** Descriptive statistics of antibody levels.

	Screening, median (log) [IQR] (n=149)	6-month follow-up, median (log) [IQR] (n=136)	8-month follow-up, median (log) [IQR] (n=94)	14-month follow-up, median (log) [IQR] (n=23)
Spike	4.28 [0.76]	3.91 [0.79]	3.79 [0.70]	3.69 [0.67]
Spike-RBD	4.19 [0.72]	3.66[0.78]	3.52 [0.62]	3.37 [0.78]
Nucleocapsid^a^	4.13 [0.78]	3.51 [0.75]	3.06 [0.71]	2.90 [0.96]
N-RBD	3.88 [0.89]	3.16 [0.93]	3.00 [0.61]	2.80 [0.88]

a Screening n=96; 6-month n=88 and 8-month n=88.

Measurements of anti-Nucleocapsid (**Figure 1D**) and anti-N-RBD (**Figure 1E**) antibodies also showed persistence of these responses with median log endpoint titers of 2.69 [IQR 0.96] and 2.8 [IQR 0.88], respectively (**Table 3**). The humoral responses to Nucleocapsid remained lower than those to Spike, as we reported previously (1). Like for Spike, we found a continued strong correlation (Spearman r=0.79, p<0.0001) of antibodies recognizing Nucleocapsid and N-RBD (**Figure 1F**).

Comparative analysis of antibody levels at month 14 were also performed taking into consideration gender, age, and hospitalization. Of these parameters, only hospitalization showed significantly higher antibody levels for both Spike (p=0.015, Mann-Whitney) and Nucleocapsid (p=0.004, Mann-Whitney) values (**Figure 1G**). These data corroborated our previous findings over the 8-month follow-up (1), supporting that hospitalization in this cohort, i.e. severity of COVID-19, has been a key factor contributing to the initial and continued magnitude of SARS-CoV-2 antibodies.

We previously reported a bi-phasic decline of the antibodies with a shorter half-life (47-97 days) between 0-6 months post symptom onset and a longer half-life between 6 to 8 months (1). We re-evaluated this assessment considering the addition of the 14-month data set (**Table 4**). These data further support our initial observation of a bi-phasic antibody decline. Antibody durability analysis showed trend of further increased half-life of Spike antibodies and a significant increase of the half-life of Nucleocapsid antibodies; and continued presence of higher Spike than Nucleocapsid antibodies also at the 14-month follow-up. The small number of patients monitored at the 14-month time point likely impacted the significance of some of the half-life measurements. Nevertheless, the continued monitoring using the same methodology strongly supports the key differences in Spike and Nucleocapsid antibody biology and points to strong durability of anti-Spike antibody when induced upon SARS-CoV-2 infection.

**Table 4:** Median slope distributions of the antibody levels.

	Month	N	Median slope	95% CI lower	95% CI upper	p^1^	Half-life, month	p^2^
**Spike**	0-6	136	-0.093	-0.104	-0.080	**<0.0001**	3.2	
	6-8	81	-0.035	-0.058	-0.018	**<0.0001**	8.6	**<0.0001**
	8-14	22	-0.011	-0.029	0.007	**0.038**	26.2	0.065
**Spike-RBD**	0-6	136	-0.128	-0.149	-0.109	**<0.0001**	2.4	
	6-8	81	-0.053	-0.066	-0.038	**<0.0001**	5.7	**<0.0001**
	8-14	22	-0.023	-0.038	-0.009	**0.0033**	13.0	0.46
**Nucleocapsid**	0-6	87	-0.188	-0.209	-0.174	**<0.0001**	1.6	
	6-8	71	-0.103	-0.128	-0.085	**<0.0001**	2.9	**<0.0001**
	8-14	22	-0.040	-0.064	-0.018	**0.0004**	7.4	**0.0092**
**N-RBD**	0-6	136	-0.190	-0.209	-0.175	**<0.0001**	1.6	
	6-8	81	-0.078	-0.098	-0.055	**<0.0001**	3.9	**<0.0001**
	8-14	22	-0.043	-0.059	-0.020	**<0.0001**	7.0	0.16

^1^H0 (=Null hypothesis): Median slope=0; ^2^H0: Equal to slope in preceding interval. Significant p values are given in bold.

### Breadth of Anti-Spike Antibodies at 14-Month Post-Infection

We examined the magnitude and breadth of anti-Spike antibody responses comparing the early (median 2 months) and late (median 14 months) phase post infection using an in-house ELISA (1, 29) in the 22 patients. Antibody titers were determined against WA1 and three variant Spike-RBD proteins including Alpha (B.1.1.7), Beta (B.1.351), Delta (B.1.617.2) and Mu (B.1.621). The RBD of Alpha, Beta and Mu share N501Y, both Beta and Mu share E484K, Beta has in addition K417N, whereas Mu has in addition R346K; Delta has L452R and T478K (**Figure 2A**).

We found strong recognition of the different variant Spike-RBD proteins at the early and late time points (**Figure 2B**). Due to natural contraction of the humoral immune responses over time, the titers were significantly lower at the late time point. WA1-induced antibodies potently recognize Alpha Spike-RBD with a magnitude similar to WA1, indicating that the N501Y change did not affect the binding capacity in the ELISA assay. Binding to Delta Spike-RBD was slightly lower (2.1-fold and 1.4-fold, respectively, at 2- and 14-month) but did not reach significance (ANOVA, Friedman test) although the differences to WA1 were significant using t test (Wilcoxon; p=0.0007 and 0.0001, respectively). On the other hand, compared to WA1, binding to Mu Spike-RBD and Beta Spike-RBD were significantly lower (ANOVA). We found lower binding to Mu at the 2- and 14-month follow-up (median 3.3- and 2.6-fold, respectively) and an even lower binding to Beta (10.6- and 5.4-fold, respectively).

These data further point the important contribution of distinct AA changes in RBD of some Spike variant (**Figure 2A**). The single AA change in Alpha (N501Y) had no effect but the combination with additional AA changes in Beta RBD (K417N and E484K) and in Mu (R346K and E484K) resulted in significant reduction in binding. On the other hand, changes in Delta-RBD (L452R and T478K) had a lesser effect. Thus, the anti-Spike antibodies induced upon infection by WA1 strain showed robust cross-reactivity among the panel of Spike-RBD tested and they maintain a similar ranking in recognition of variant Spike-RBD at early (2 months) and late (14 months) time points post symptom onset.

### Durability of Neutralizing Antibody at 14-Month Post-Infection

**Figure 3:**
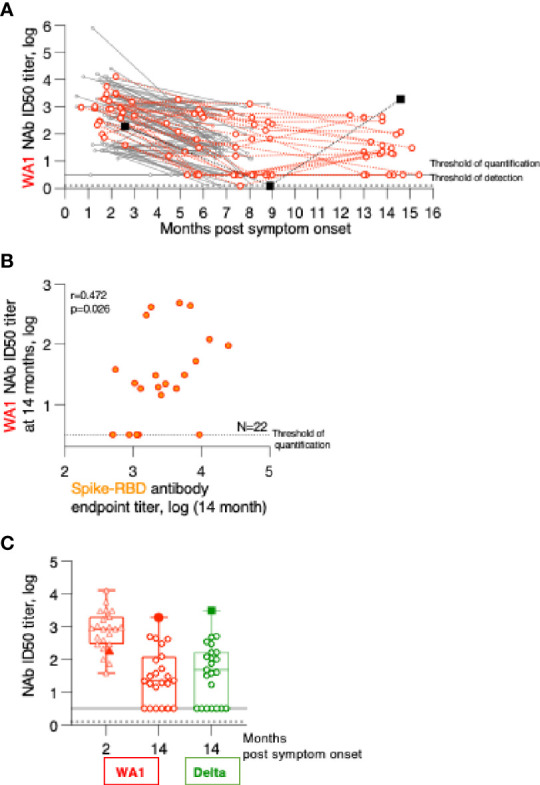
Persistence of Neutralizing Antibodies (NAb) responses. **(A)** WA1 neutralizing antibodies were measured using the pseudotype SARS-CoV-2 virus inhibition assay and the ID50 (log) values were plotted over time. **(B)** Correlation of WA1 Spike-RBD and NAb measured at month 14. Spearman R and values are given. **(C)** WA1 and Delta NAb were measured in the 23 patients at the 2- and the 14-month follow-up using a pseudotyped HIV_NL_ΔEnv-Nanoluc assay carrying the indicated Spike (AA 1-1254) proteins. The ID50 (log) of the neutralization curves were plotted. Threshold of detection in gray solid line; threshold of quantification in grey dotted line.

We compared the neutralizing capabilities of the anti-Spike antibodies. **Figure 3A** shows the previously reported patients up to the 8-month time point with an overlay of the 23 patients sequentially analyzed over the 14-month follow-up. We found that 6 of the 22 samples were below threshold of the assay, in agreement with the observation up to the 8-month time point (1) where we reported that 24% of convalescent patients failed to show detectable NAb in this assay.

The relation of the Spike-RBD antibodies and their ability to neutralize WA1 Spike pseudotyped virus at 14 months post symptom onset was examined (**Figure 3B**). This data showed that our previously reported correlations for the analysis between the 2- to 8-month follow-up (1) are maintained (Spearman r= 0.472, p=0.024). The 14-month comparison also showed a stronger contraction of NAb reaching the threshold level of the assay despite detectable ELISA responses.

Comparison to the peak NAb responses showed a 7-fold reduction in inhibition of WA1 pseudotype virus infection over time (**Figure 3C**). Testing of the NAb breadth showed robust neutralization of Delta (**Figure 3C**) reaching similar levels to those of WA1 at the 14-month time point.

### Convalescent Patient With SARS-CoV-2 Re-Exposure, a Case-Report

**Figure 4:**
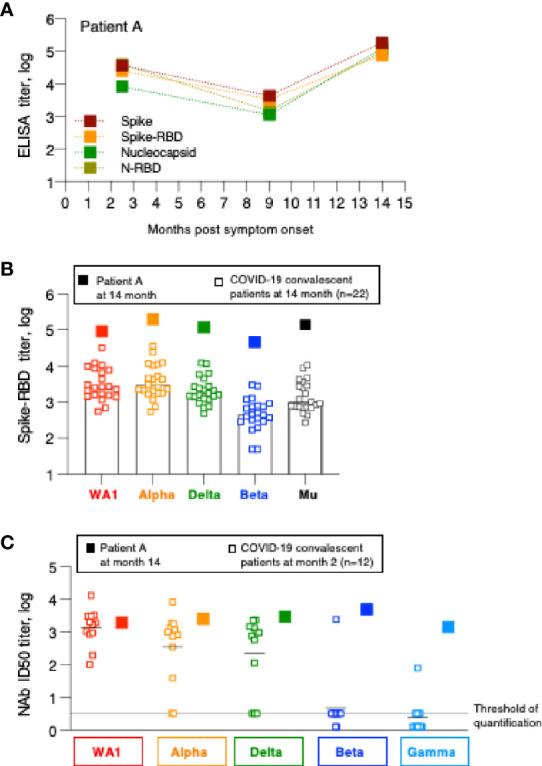
Humoral immune response analysis of patient A. **(A)** Antibody endpoint titers of patient A were measured at 2, 9 and 14 months post symptom onset against trimeric Spike, Spike-RBD, complete Nucleocapsid and N-RBD (data are from **Figure 1**). **(B)** Antibody endpoint titers from patient A (filled squares) were determined against a panel of variant Spike-RBD proteins. The data are superimposed on the data obtained from the SARS-CoV-2 convalescent patients shown in **Figure 2**. **(C)** NAb of patient A at the 14-month follow-up were measured against a panel of pseudotyped viruses carrying the indicated Spike proteins. The data are compared with the NAb ID50 obtained from SARS-CoV-2 convalescent patients (n=22) shown in **Figure 3**, measured at the 2-month follow-up.

Humoral immune response analysis (**Figure 1**) showed that in contrast to the other patients, one patient showed sharp increase in antibodies between month 9 and 14 (summarized in **Figure 4A**). This patient (referred to as patient A) is a 43-year old male who had reported SARS-CoV2 symptoms on 3/15/2020, tested positive by PCR on 3/16/2020 and was hospitalized at this time and was monitored over time as part of this study. Over the course of the follow-up, he showed antibody specificities, magnitude, and contraction of responses, similarly to the other patients in this cohort (**Figure 1**). We noted that at the 9-month follow-up, he did not have any detectable NAb (**Figure 3**). Interestingly, between month 9 and 14, patient A showed sharp increases in Spike, Spike-RBD as well as Nucleocapsid and N-RBD antibody responses (**Figure 1** **;** **Figure 4A**). These data demonstrate re-exposure to SARS-CoV-2 with strong anamnestic responses to both Spike and Nucleocapsid. Of note, patient A’s antibody endpoint titers were very high with a magnitude reached only by few patients in the very early phase (1-2 months post symptom onset; **Figure 1**) (1, 2, 14), indicating a very strong recall response. Patient A’s antibodies also potently recognized the Spike-RBD of the Alpha, Beta, Delta and Mu variants (**Figure 4B**). Although this is a single case, we noted that the recognition of WA1, Alpha and Mu was of similar magnitude and Beta was slightly lower, whereas the cohort of the other convalescent patients (**Figure 2**) showed significantly impaired binding to both Mu and Beta RBD.

Patient A also showed a robust increase in WA1-specific NAb responses which were at the threshold of detection at month 9 and increased to highest levels at month 14 (**Figure 3**). The antibodies showed good cross-neutralization of Delta and Alpha (**Figures 3B**, **4C**). Interestingly, we noted that patient A’s NAb showed stronger inhibition of Beta and Gamma relative to WA1 and compared to the responses measured in convalescent patients from this cohort early post symptom onset (**Figure 4C**). The Beta and Gamma variants share the same AA changes in RBD but differ by several additional AA changes in Spike.

In conclusion, although patient A did not report any signs of recurrence of COVID-19 infection, changes in both Spike and Nucleocapsid antibody levels demonstrate that he was re-exposed between December 2020 and May 2021 but due the high magnitude of the humoral immune responses it is more likely that this occurred in Spring 2021. The increased Spike-RBD binding antibody as well as increased neutralization breadth suggest that re-exposure to the virus resulted either in a significant increase in magnitude and breadth or that the individual was exposed to a new SARS-CoV-2 variant, providing better cross-reactivity to Beta and Gamma.

## Discussion

In this report, we present a longitudinal humoral immune response analysis of SARS-CoV-2 convalescent patients who were monitored as early as 2 months post symptom onset to the 14-month follow-up. These data are part of our previously reported 148-patient evaluation using the same methodologies (1, 2). We measured Spike and Nucleocapsid antibody responses over time in a subset of 23 patients, who remained unvaccinated. All patients in our cohort remained positive for anti-SARS-CoV-2 antibodies. However, several convalescent patients (9 of 22) decreased neutralizing Ab titers below the threshold of the assay, as also anticipated from the 8-month follow-up, where we reported that 24% of the patients reduced NAb to undetectable levels (1).

We previously reported a bi-phasic decline with an inclination point at ~6 months post symptom onset (1). The short half-lives of Spike and Nucleocapsid antibodies during the first 6 months post symptom onset were followed by longer half-lives (month 6-8) for both specificities. The continued monitoring of a subset of patients allowed us to further compare the durability of the antibody responses between month 8 and 14. We found that the previously reported elongation of half-lives in the ‘late’ phase post-infection continued. Thus, after an initial reduction of the respective half-lives, both Spike and Nucleocapsid antibodies show elongated half-lives.

We found that anti-Spike antibodies show a strong ability to recognize and neutralize the autologous WA1, as well as the Alpha and Delta Spike variants, but that they were greatly impaired in recognizing the Beta and the Mu variants. These data are in agreement with recent reports analyzing WA1-derived vaccine-induced immune responses to Alpha, Beta and Delta (29, 33–39) which did not assess ability to bind to the recently identified Mu variant. These data point to the importance of single AA changes within RBD, which may impair recognition and severely dampen the neutralization capabilities of the antibodies. Recent emerging Variants of Concern show a multitude of such changes which will likely affect NAb function of the WA1-induced antibodies. We and others reported that WA1 antibody showed lower binding to Delta both in humans (29, 33–38) and vaccinated macaques (32).

Importantly, our data further showed that SARS-CoV-2 infection-induced antibodies show strong neutralization of Delta, which is the driver strain of the present epidemic. The antibodies are maintained for over 14 months post symptom onset with a ranking of Alpha>Delta>Mu>Beta. Binding antibody strength is predictive of its neutralization capability (29, 32), supported by stronger NAb against Delta compared to Beta. We also found that the neutralization capability of SARS-CoV-2 infection-induced antibodies against the Delta variant is better than the BNT162b2 mRNA vaccine induced responses (29). These findings point to important differences in antibody development due to antigen exposure upon infection.

An important aspect of monitoring humoral responses has been having access to plasma from the same patients over 14 months. Most reports were based on cross-sectional analysis spanning less than 6 months (3–15) or up to 12 months (1, 4, 16–27). Depending on the patient cohort, several studies reported a stronger initial contraction (1, 4, 23–25, 27) of anti-COVID humoral responses. Our longitudinal study spanning 2-14 months post symptom onset allowed for more detailed estimation of half-live calculations. We reported a bi-phasic decline with an inclination point at 6-month post symptom onset (1). The shorter half-lives of Spike and Nucleocapsid antibodies during the first 6 months were followed by longer half-lives for both specificities up to the 8-month analysis (1). The 14-month analysis further supported our initial observation of a bi-phasic antibody decline with increased half-lives and continued presence of both Spike and Nucleocapsid antibodies after the inclination point. Thus, monitoring durability of SARS-CoV-2 vaccine recipients will allow future comparisons between infection- and vaccination-induced anti-Spike antibody longevity.

An interesting observation has been the immune response changes in Patient A. This is a single case in our longitudinal study, who presumably was re-exposed to SARS-CoV-2 but did not develop any COVID-19-related symptoms. The increase in antibodies against nucleocapsid protein agrees with the conclusion that this person was exposed to the whole virus and boosted all virus specific antibodies tested. Patient A possibly was re-exposed in the period of Spring 2021. This patient represents an example of strong protective immunity despite his very low circulating NAb being below the detection threshold at the 9-month follow-up, the last time point analyzed before his re-exposure. Therefore, study of similar patients may provide an opportunity to define a level of circulating antibodies able to protect from symptomatic disease after re-infection. In such a case, we would conclude that circulating antibody log10 titers of 3.5 for Spike, using our in-house ELISA, protect from disease development, despite below threshold levels of circulating NAb. Alternatively, such protection may depend not on circulating antibodies but on the ability for a rapid and efficient recall response and/or possible additional antibody effector functions (40). As mentioned above, this patient had NAb below the lower level of detection of the present assay at the 9-month follow-up, although they were detectable during the first period after infection.

Breakthrough infections occur in individuals with vaccine- or infection-induced SARS-CoV-2 immunity, especially by the circulating Delta strain (41–43). Such infections may be more frequent or more pathogenic in individuals with low anti-WA1 antibodies. Therefore, further analysis and identification of protective immune response is essential for the overall defense against the current pandemic.

We conclude that all 23 convalescent patients with COVID-19 continued to have antibodies able to recognize a panel of Spike proteins and 60% of these patients have NAbs against the WA1 and the Delta variant of SARS-CoV-2, suggesting a strong long-lasting response that may offer a prolonged protection against re-infection. The confirmation of these results in a larger cohort of patients will provide important information for the best time of vaccination for these individuals.

## Data Availability Statement

The original contributions presented in the study are included in the article/supplementary material. Further inquiries can be directed to the corresponding authors.

## Ethics Statement

This study included plasma donors who participated in a phase 2 study (NCT04408209 and NCT04743388) for the use of convalescent plasma for the treatment of COVID-19 infection, started in Greece on April 28, 2020. All study procedures were carried out in accordance with the declaration of Helsinki (18th World Medical Association Assembly), its subsequent amendments, the Greek regulations and guidelines, as well as the good clinical practice guidelines (GCP) as defined by the International Conference of Harmonization. The study was also approved by the local ethics committees of all participating hospitals. The patients/participants provided their written informed consent to participate in this study.

## Author Contributions

ET, M-AD, BF, and GP conceived and designed the study. IN and EK collected and processed patient data and samples. MR, MA, JB, RB, XH, DD, and DV performed data analysis. MR, GP and BF verified the underlying data. MR, EV, GP, and BF drafted the manuscript. All authors critically revised the manuscript for important intellectual content and gave final approval for the submitted version.

## Funding

This work was supported by funds from the Intramural Research Program, National Institutes of Health, National Cancer Institute, Center for Cancer Research to GP and BF.

## Conflict of Interest

The authors declare that the research was conducted in the absence of any commercial or financial relationships that could be construed as a potential conflict of interest.

## Publisher’s Note

All claims expressed in this article are solely those of the authors and do not necessarily represent those of their affiliated organizations, or those of the publisher, the editors and the reviewers. Any product that may be evaluated in this article, or claim that may be made by its manufacturer, is not guaranteed or endorsed by the publisher.
